# Sequential high-dose cytarabine and mitoxantrone (S-HAM) versus standard double induction in acute myeloid leukemia—a phase 3 study

**DOI:** 10.1038/s41375-018-0268-9

**Published:** 2018-10-01

**Authors:** Jan Braess, Susanne Amler, Karl-Anton Kreuzer, Karsten Spiekermann, Hans Walter Lindemann, Eva Lengfelder, Ullrich Graeven, Peter Staib, Wolf-Dieter Ludwig, Harald Biersack, Yon-Dschun Ko, Michael J. Uppenkamp, Maike De Wit, Stefan Korsten, Rudolf Peceny, Tobias Gaska, Xaver Schiel, Dirk M. Behringer, Michael G. Kiehl, Bettina Zinngrebe, Gerald Meckenstock, Eva Roemer, Dirk Medgenberg, Ernst Spaeth-Schwalbe, Gero Massenkeil, Heidrun Hindahl, Rainer Schwerdtfeger, Guido Trenn, Cristina Sauerland, Raphael Koch, Martin Lablans, Andreas Faldum, Dennis Görlich, Stefan K. Bohlander, Stephanie Schneider, Annika Dufour, Christian Buske, Michael Fiegl, Marion Subklewe, Birgit Braess, Michael Unterhalt, Anja Baumgartner, Bernhard Wörmann, Dietrich Beelen, Wolfgang Hiddemann, for the AML-CG

**Affiliations:** 1 Department of Oncology and Hematology, Hospital Barmherzige Brüder, Regensburg, Germany; 2 Department of Medicine III, University Hospital LMU Campus Grosshadern, Munich, Germany; 3 Insitute for Biostatistics and Clinical Research, University Hospital, Münster, Germany; 4 Friedrich Löffler Institute, Federal Research Centre, Greifswald-Insel Riems, Germany; 5 Department of Internal Medicine I, University Hospital, Cologne, Germany; 6 Department of Hematology and Oncology, Catholic Hospital, Hagen, Germany; 7 Department of Medicine III, University Hospital, Mannheim, Germany; 8 Department of Medicine I, Hospital Maria Hilf, Mönchengladbach, Germany; 9 Department of Hematology and Medical Oncology, St. Antonius Hospital, Eschweiler, Germany; 10 Department of Hematology and Oncology and Tumor Immunology, Helios Hospital, Berlin-Buch, Germany; 11 Department of Medicine I, University Hospital, Lübeck, Germany; 12 Department of Medicine I, Johanniter Hospital, Bonn, Germany; 13 Department of Medicine A, Klinikum Ludwigshafen, Ludwigshafen, Germany; 14 Department of Hematology, Oncology and Palliative Care, Vivantes Klinikum Neukölln, Berlin, Germany; 15 Department of Medicine, Vinzenz Pallotti Hospital, Bergisch-Gladbach, Germany; 16 Department of Hematology and Oncology, Klinikum Osnabrück, Osnabrück, Germany; 17 Department of Hematology and Oncology, St. Josef Hospital, Paderborn, Germany; 18 Department of Hematology and Oncology, Klinikum Harlaching, Munich, Germany; 19 Department of Hematology, Oncology and Palliative Care, Augusta Hospital, Bochum, Germany; 20 Department of Medicine I, Klinikum Frankfurt/Oder, Frankfurt/Oder, Germany; 21 Department of Hematology, Oncology and Palliative Care, Evangelisches Krankenhaus, Bielefeld, Germany; 22 Department of Medical Oncology, Radiooncology, Hematology and Palliative Care, St. Josef Hospital, Gelsenkirchen, Germany; 23 Department of Hematology and Oncology, Klinikum Idar-Oberstein, Idar-Oberstein, Germany; 24 Department of Medicine III, Klinikum Leverkusen, Leverkusen, Germany; 25 Department of Medicine, Vivantes Klinikum Spandau, Berlin, Germany; 26 Department of Medicine II, Klinikum Gütersloh, Gütersloh, Germany; 27 Department of Medicine I, St. Johannes Hospital, Dortmund, Germany; 28 Department for Bone Marrow and Blood Stem Cell Transplantation, DKD Deutsche Klinik für Diagnostik, Wiesbaden, Germany; 29 Department of Medicine I, Knappschaftskrankenhaus, Bottrop, Germany; 30 Division of Medical Informatics in Translational Oncology, DKFZ German Cancer Research Center, Heidelberg, Germany; 31 Department of Molecular Medicine and Pathology, University of Auckland, Auckland, New Zealand; 32 Institute of Experimental Cancer Research, University Hospital, Ulm, Germany; 33 German Society of Hematology and Oncology DGHO, Berlin, Germany; 34 Department of Bone Marrow Transplantation, University Hospital, Essen, Germany

## Abstract

Dose-dense induction with the S-HAM regimen was compared to standard double induction therapy in adult patients with newly diagnosed acute myeloid leukemia. Patients were centrally randomized (1:1) between S-HAM (2nd chemotherapy cycle starting on day 8 = “dose-dense”) and double induction with TAD-HAM or HAM(-HAM) (2nd cycle starting on day 21 = “standard”). 387 evaluable patients were randomly assigned to S-HAM (*N* = 203) and to standard double induction (*N* = 184). The primary endpoint overall response rate (ORR) consisting of complete remission (CR) and incomplete remission (CR_i_) was not significantly different (*P* = 0.202) between S-HAM (77%) and double induction (72%). The median overall survival was 35 months after S-HAM and 25 months after double induction (*P* = 0.323). Duration of critical leukopenia was significantly reduced after S-HAM (median 29 days) versus double induction (median 44 days)—*P* < 0.001. This translated into a significantly shortened duration of hospitalization after S-HAM (median 37 days) as compared to standard induction (median 49 days)—*P* < 0.001. In conclusion, dose-dense induction therapy with the S-HAM regimen shows favorable trends but no significant differences in ORR and OS compared to standard double induction. S-HAM significantly shortens critical leukopenia and the duration of hospitalization by 2 weeks.

## Introduction

Even though treatment of acute myeloid leukemia (AML) with curative intent is not standardized worldwide, it always involves intensive induction chemotherapy with the aim of reaching a (morphologically) complete remission (CR) and subsequent risk stratified post-remission therapy. Except for acute promyelocytic leukemia (APL) [1] and—recently—FLT3 mutated AML [2], the substantial (genetic) heterogeneity of AML [3, 4] so far has had only a moderate impact on the choice of therapy. Specifically, the increased knowledge of AML biology has not yet obviated the necessity for intensive cytotoxic chemotherapy. This is especially true for the initial induction phase where leukemia burden reduction by 3–4 logs and achievement of a complete (morphological) remission—following a prolonged period of deep aplasia—is the main goal.

One current standard induction therapy approach—as established by the AML-CG and many other cooperative groups in the last decades—is “double induction” (DI) therapy [5–8]. This strategy consists of two cycles of intensive cytarabine (AraC) and anthracycline/anthracenedione-based chemotherapy. In younger patients (<60 years), the second chemotherapy cycle is given mandatorily on day 21 even if no residual blasts are detected in a bone marrow aspirate on day 16 (“mandatory DI”). In older patients (≥60 years), the second cycle is only given in case of residual blasts at this time point (“conditional DI”). In an attempt to further improve this approach, a time-sequential modification of the high-dose AraC/mitoxantrone combination (S-HAM) was developed into a dose-dense application of high-dose AraC (HD-AraC) on days 1 and 2 followed by mitoxantrone on days 3 and 4 and a repetition of the same sequence after a 3-day treatment-free period from days 8 to 11. Hence, the S-HAM regimen covers a total of 11 days of treatment only. With this regimen, highly encouraging results were obtained for salvage therapy in relapsed and refractory AML [9]. Hence, the AML-CG explored this dose-dense concept subsequently in first-line therapy [10]. The AML-CG 2004 pilot trial demonstrated the feasibility and high efficacy of the S-HAM regimen without increasing toxicity and revealed a substantial reduction in the duration of critical leukopenia. The current AML-CG 2008 study was initiated to confirm these results and to compare the efficacy and toxicity of S-HAM with standard double induction within a prospective randomized trial using the overall response rate (ORR) as the primary endpoint.

Secondary endpoints were overall survival (OS), the duration of critical cytopenias, and the duration of hospitalization, amongst others.

## Patients and methods

### Study conduct

The current study was carried out in accordance with the declaration of Helsinki. All patients gave their consent after having been informed about the purpose and the investigational nature of the trial. Before initiation, the study received approval of the responsible institutional review board and the ethics committees of the participating institutions. The clinical study is an official study of the “Kompetenznetz Akute und Chronische Leukämien” and is registered in the European Trial Registry as EudraCT 2007-003103-12.

### Patients

Patients aged ≥18 years with newly diagnosed AML including de-novo AML and secondary AML after a preceding hematological disorder could be included. Patients with APL were excluded. There was no upper age limit, but patients needed to be considered fit for intensive AML therapy by their treating physician.

### Induction therapy

For induction therapy, eligible patients were randomly assigned to standard double induction or S-HAM with stratifications for de novo or secondary AML and age <60 or ≥60 years. Within the group of patients between 60 and 70 years of age, a distinction was made according to biologic rather than chronologic age, i.e., patients of ages 60–70 years who were considered biologically “young” were included into the “young” population while biologically “old” patients were included into the “old” treatment group. This discrimination was applied for the selection of standard induction which was TAD-HAM in younger patients and HAM-(HAM) in older patients, and was compared to S-HAM as the experimental arm. The allocation to the “young” or “old” group was done at the discretion of the treating physician and was performed at registration/randomization. Please note that only chronological age was used for dose determination in HD-AraC components—i.e., 3 g/m^2^ for patients <60 years and 1 g/m^2^ for patients 60+ years.

#### Standard double induction in younger patients

Standard double induction in younger patients consisted of TAD 9 followed by HAM on day 21. The TAD-9 regimen comprised a continuous infusion of AraC at 100 mg/m^2^ per day for the first 48 h followed by short infusions of AraC (100 mg/m^2^) twice daily on days 3–8. Daunorubicin was applied on days 3–5 at 60 mg/m^2^ as a 1 h infusion and thioguanine was given orally (100 mg/m²) twice daily on days 3–9. Seven days after the completion of the first cycle (i.e., on day 16 after the start of TAD-9), a bone marrow aspirate was taken to evaluate the degree of leukemic cytoreduction. The second cycle of double induction was started on day 21 irrespective of peripheral blood counts and irrespective of the blast count in the bone marrow aspirate on day 16 (“mandatory” DI). For the second cycle, the HAM regimen was applied consisting of HD-AraC at 3 g/m^2^ (1 g/m^2^ for patients 60+ years chronological age) as a 3 h infusion every 12 h on days 1–3. Mitoxantrone was applied at a dose of 10 mg/m^2^ as 1 h infusion on days 3–5.

#### Standard induction in older patients

Standard induction in older patients consisted of HAM followed by the second HAM cycle on day 21 only in case of an inadequate leukemic cytoreduction (>5% residual blasts in the bone marrow smears) after the first HAM cycle (“conditional” DI).

#### The S-HAM regimen

The S-HAM regimen consisted of HD-AraC at a dose of 3 g/m^2^ (1 g/m^2^ for patients age 60+ years chronological age) as a 3 h infusion every 12 h on days 1–2 and days 8–9. Mitoxantrone was applied at a dose of 10 mg/m^2^ as a 1 h infusion on days 3–4 and 10–11. Two to seven days after completion of the S-HAM regimen (i.e., on days 13–18 after the start of S-HAM), a bone marrow aspirate was obtained to determine the degree of leukemic cytoreduction.

**Fig. 1:**
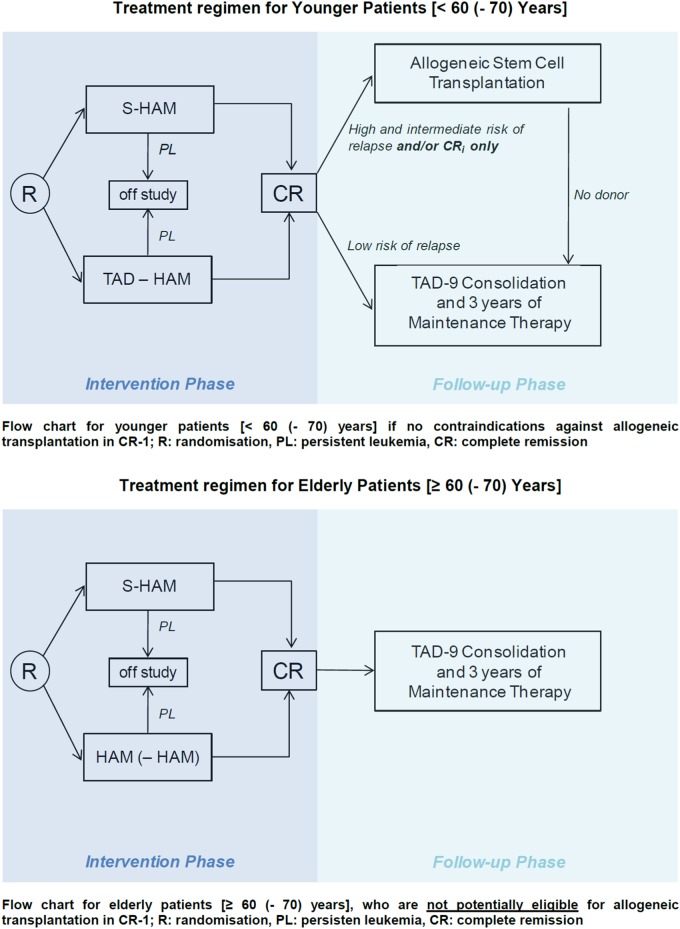
Flow chart of protocol treatment

The flow chart of the study is depicted in Fig. 1.

### Supportive care during induction

Standard supportive measures were applied including antifungal prophylaxis with posaconazole. Growth factor support after the completion of induction therapy was mandatory after S-HAM with pegylated G-CSF (6 mg s.c.) on day 18 if there were no residual blasts in the posttreatment bone marrow aspirate. After standard double induction, G-CSF application was optional if no residual blast had been observed in the day 16 bone marrow aspirate. This was also possible in “older” patients receiving the second cycle due to prior insufficient blast clearance after the first cycle.

### Postremission therapy

Patients in CR after induction therapy were placed on consolidation treatment with one cycle of TAD and 3 years of monthly myelosuppressive maintenance with alternating 5-day cycles of AraC plus thioguanine, daunorubicin, or cyclophosphamide (AD-AT-AD-AC…) [11, 12] in patients considered to be at a low risk of relapse or not eligible for allogeneic transplantation. Eligible patients with a high or intermediate risk of relapse were allocated to allogeneic transplantation in first remission (CR-1).

The formal interventional phase of the present study involved only the induction period until the response to treatment could be evaluated with a maximum of 90 days after the start of induction treatment. Postremission treatment was defined but was not part of the formal study.

### Endpoints and definitions

The primary endpoint of the study was the ORR comprizing CR and CR_i_ (complete remission with incomplete peripheral recovery) [13] after the completion of induction treatment. CR was documented after normalization of peripheral blood counts by a bone marrow aspirate immediately prior to consolidation therapy. Secondary endpoints included OS, event-free survival (EFS) and relapse-free survival (RFS), non-hematological toxicities and hematological toxicities during induction, duration of critical cytopenia, and duration of hospitalization. These secondary endpoints were exploratory in nature and were not powered to be definitive.

### Statistics

The trial addressed the hypothesis that the ORR might be improved by S-HAM by 15% from 70% as expected for standard double induction to 85% for S-HAM. The primary confirmatory endpoint was analyzed using a sequential one-sided truncated probability ratio test [14]. The significance level was set to 0.05. Based on these assumptions, 360 evaluable patients had to be recruited to achieve a power of 95% (see study protocol). Patient characteristics between the two randomized treatment regimens were evaluated using Fisher’s exact test for categorical and Wilcoxon rank-sum test for continuous variables with the respective 95% confidence intervals (95% CI). Categorical variables are reported as absolute and relative frequencies. Continuous variables are shown as median [minimum − maximum]. Time-to-event outcomes were analyzed by two-sided log-rank tests. Kaplan–Meier method was used to estimate event rates and 95% confidence limits (95% CI). Duration of critical cytopenia was calculated from the start of therapy until recovery of peripheral blood counts and was presented as inverse Kaplan–Meier curves. Primary and secondary efficacy analyses were performed on an intention-to-treat basis. All secondary and subgroup analyses have to be considered exploratory and thus no adjustment for multiplicity was performed. *P*-values ≤ 0.05 were considered as significant. The primary analysis (sequential test) was performed using the SAS software (SAS® software, version 9.2, for Windows (SAS Institute, Cary, NC, USA)). All other statistical analyses were performed using the SAS^®^ software version 9.4.

## Results

### Patient characteristics

**Fig. 2:**
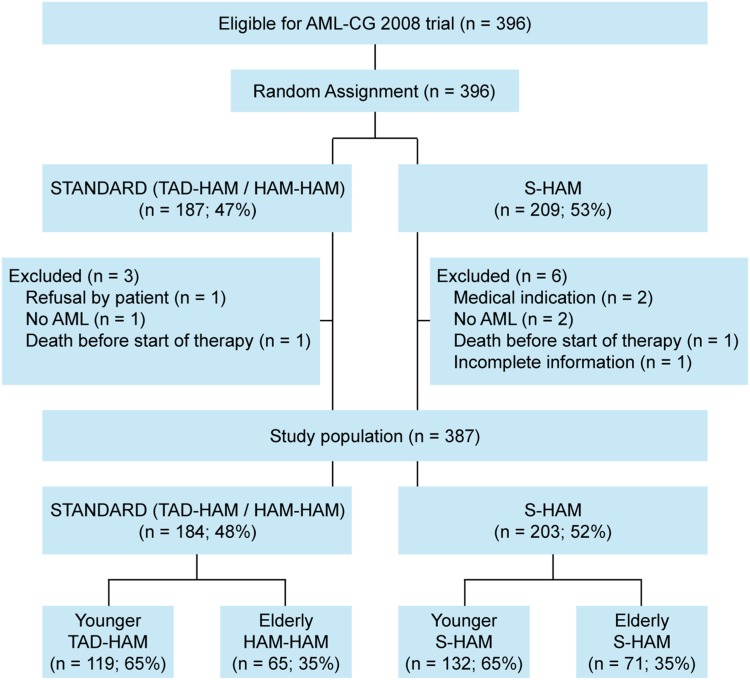
Study flow diagram (CONSORT)

From July 2009 until March 2012, a total of 396 patients were randomized into the study. After exclusion of nine patients, 387 evaluable patients were compared between S-HAM [*n* = 203 (52%)] and standard double induction [*n* = 184 (48%)] (Fig. 2). Patient characteristics were similar between both treatment groups (Table 1). The median age was 58 years, 46% of patients had a chronological age ≥60 years. The group of “younger” patients including patients between 60 and 70 years who were considered biologically young consisted of 251 (65%) patients and was randomized between S-HAM and (mandatory) DI with TAD-HAM. Correspondingly, 136 (35%) patients belonged to the “older” age group ≥60 and were randomized between S-HAM and (conditional) DI with HAM(-HAM). Compliance to the assigned therapy was 99% in both the treatment groups.

**Table 1:** Patient characteristics by randomization

Treatment arm	All randomized and evaluable patients in the study		
	Standard	S-HAM	*P*
No. of patients, *N* (%)	184 (48)	203 (52)	
Age, years			0.722^a^
Median (range)	58 (18–86)	58 (19–81)	
Sex			0.026^b^
Female/male, *N* (%)	76/108 (41/59)	107/96 (53/47)	
Patient age (chronological), *N* (%)			0.760^b^
<60 years	101 (55)	108 (53)	
≥60 years	83 (45)	95 (47)	
Patient age (biological), *N* (%)			
Younger: <60 (−70)	119 (65)	132 (65)	
Median (range)	51 (18–68)	52 (19–70)	0.815^a^
Older: ≥60 (−70)	65 (35)	71 (35)	
Median (range)	68 (60–86)	68 (57–81)	0.101^a^
AML subtype, *N* (%)			0.091^b^
de novo AML	143 (78)	154 (76)	
AML secondary to MDS (s-AML)	22 (12)	37 (18)	
AML therapy-related (t-AML)	19 (10)	12 (6)	
Blasts in bone marrow, %			0.551^a^
Median	65	68	
Range	10‒97	6‒100	
LDH, U/L			0.721^a^
Median (range)	334 (107–4833)	356 (117–3431)	
ECOG, *N* (%)			0.631^b^
0	65 (36)	62 (31)	
1	94 (52)	107 (53)	
2	17 (9)	23 (11)	
3	5 (3)	7 (4)	
4	0	2 (1)	
Missing/unknown	3	2	
Karyotype, *N* (%)			0.006^b^
Favorable	15 (9)	5 (3)	
Intermediate	104 (65)	146 (78)	
Unfavorable	41 (26)	37 (20)	
Missing/unknown	24	15	
Molecular aberrations, *N* (%)			0.178^b^
NPM1			
Pos.	53 (33)	74 (41)	
Neg.	106 (67)	107 (59)	
Missing/unknown	25	22	
FLT3-ITD			0.895^b^
Pos.	34 (21)	38 (21)	
Neg.	125 (79)	146 (79)	
Missing/unknown	25	19	
FLT3-TKD			1.000^b^
Pos.	11 (8)	10 (8)	
Neg.	120 (92)	121 (92)	
Unknown	53	72	
MLL-PTD			0.554^b^
Pos.	11 (7)	17 (9)	
Neg.	143 (93)	164 (91)	
Missing/unknown	30	22	
CEBPA			0.676^b^
Pos.	4 (11)	2 (6)	
Neg.	32 (89)	30 (94)	
Missing/unknown	148	171	

*MDS* myelodysplastic syndrome, *LDH* lactate dehydrogenase serum level, *ECOG* Eastern Cooperative Oncology Group, *NPM1* nucleophosmin, *FLT3* fms-like tyrosine kinase 3, *ITD* internal tandem duplication, *TKD* tyrosine kinase domain, *MLL-PTD* mixed linage leukemia-partial tandem duplication, *CEBPA* CCAAT/enhancer-binding protein alpha

^a^*P*-values are from Wilcoxon rank-sum test

^b^*P*-values are from Fisher’s exact test

### Treatment response (1° endpoint) and survival (2° endpoint)

In the total group, the ORR (consisting of CR and CR_i_) was 75%, the rate of persistent leukemia (PL) was 12%, and the rate of early death (ED) was 13%. ED until day 90 was 16% (Table 2). Please note that some patients had PL as their induction result but then died later on but within 90 days after the start of treatment. These patients were also counted in the “ED until day 90”.

**Table 2:** Treatment outcome by randomization

Treatment arm	All randomized and evaluable patients in the study						
	Total patients		Standard^a^		S-HAM		
	*N* (%)	95% CI	*N* (%)	95% CI	*N* (%)	95% CI	*P*
Patients randomized							
Total	387		184		203		
Younger	251		119		132		
Older	136		65		71		
Overall response (CR + CRi)							
Total	289 (75)	70–79	133 (72)	65–79	156 (77)	70–83	0.202^b^
Younger	197 (79)	73–83	91 (76)	68–83	106 (80)	73–86	0.539^c^
Older	92 (68)	59–75	42 (65)	53–75	50 (70)	59–80	0.582^c^
Induction result							
Total							0.309^c^
CR	200 (52)	46–57	86 (47)	39–54	114 (56)	49–63	
CRi	89 (23)	19–28	47 (25)	19–32	42 (21)	15–27	
Persistent leukemia	48 (12)	9–16	24 (13)	8–19	24 (12)	7–17	
Early death	50 (13)	9–17	27 (15)	9–21	23 (11)	7–17	
Younger							0.717^c^
CR	136 (54)	47–60	60 (50)	41–60	76 (57)	48–66	
CRi	61 (24)	19–30	31 (26)	18–35	30 (23)	16–31	
Persistent leukemia	24 (10)	6–14	12 (10)	6–17	12 (9)	5–15	
Early death	30 (12)	8–17	16 (14)	8–21	14 (11)	6–17	
Older							0.438^c^
CR	64 (47)	38–56	26 (40)	28–53	38 (53)	41–65	
CRi	28 (20)	14–28	16 (25)	15–37	12 (17)	9–28	
Persistent leukemia	24 (18)	12–25	12 (18)	10–30	12 (17)	9–27	
Early death	20 (15)	9–22	11 (17)	8–28	9 (13)	6–23	
Early death until day 90							
Total	60 (16)	12–20	29 (16)	11–22	31 (15)	11–21	1.000^c^
Younger	36 (14)	11–19	16 (13)	8–21	20 (15)	10–22	0.722^c^
Older	24 (18)	12–25	13 (20)	12–31	11 (16)	9–26	0.509^c^
Overall survival							
Total							0.323^d^
Median, months	29	20–38	25	15–34	35	21–49	
Younger							0.742^d^
Median, months	48	25–71	45	16–74	48	25–71	
Older							0.219^d^
Median, months	19	12–25	19	10–27	19	11–28	
Event-free survival							
Total							0.753^d^
Median, months	11	8–13	10	6–15	11	8–14	
Younger							0.980^d^
Median, months	14	7–22	18	7–29	14	6–21	
Older							0.441^d^
Median, months	7	4–10	6	3–9	9	4–14	

*CR* complete remission, *CRi* incomplete remission

^a^Standard treatment in “younger” patients was TAD-HAM (double induction mandatory), in “older” patients it was HAM(-HAM) (double induction only conditional if no adequate blast clearance (  5%) had been achieved after one cycle of HAM)

^b^*P*-values are from one-sided sequential truncated probability ratio test (Whitehead)

^c^*P*-values are from Fisher’s exact test

^d^*P*-values are from log-rank test

In the S-HAM group, ORR was higher but not statistically significant with 77% (95% CI: 70–83%) as compared to 72% (95% CI: 65–79%) for the standard DI group (*P* = 0.202). In the “younger” group the ORR after S-HAM was 80% versus 76% after standard DI with TAD-HAM. In the “older” group the ORR after S-HAM was 70% versus 65% after standard DI with HAM(-HAM).

**Fig. 3:**
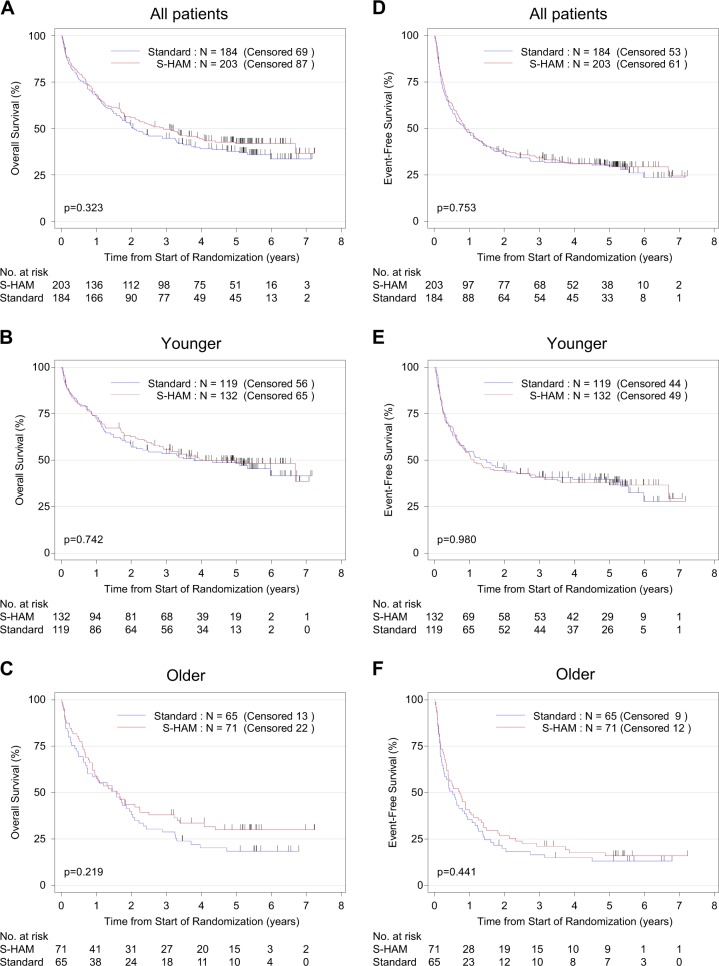
Overall survival and event-free survival. Overall survival of all patients (**a**), of patients younger than <60 years (**b**), of patients older than ≥60 years (**c**). Event-free survival of all patients (**d**), of patients younger than <60 years (**e**), of patients older than ≥60 years (**f**)—always biological age as defined in “Patients and methods”

The median follow-up was 62 months. Over all patients the median OS was 29 months. Following S-HAM treatment, the median OS was 35 months versus 25 months after standard DI (*P* = 0.323). For the age strata, the results on OS were 48 months for S-HAM versus 45 months after standard DI in the “younger” group and 19 months (S-HAM) versus 19 months (standard DI) in the “older” group (Fig. 3). There were no significant differences in EFS (also Fig. 3) and in RFS (data not shown).

No significant differences between treatment arms were observed for ORR nor OS in the prespecified karyotype subgroups (data not shown).

### Toxicities and early death (ED) rate (2° endpoints)

Non-hematological toxicities grade 3 and 4 are listed in Table 3. For the total group, the most relevant toxicities were infection 48%, pulmonary toxicity 19%, pain 16%, fever 13%, weight gain 12%, diarrhea 11%, liver toxicity 10%, nausea/vomiting 9%, and bleeding 7%. There were no statistically significant differences between the S-HAM group and the standard DI group except for bleeding, 10% (S-HAM) versus 4% (standard DI) and mucositis, 10% (S-HAM) versus 3% (standard DI).

**Table 3:** Incidence of non-hematological toxicities grade 3 and 4 by randomization

Treatment arm	All randomized and evaluable patients in the study					
	Total patients		Standard		S-HAM	
	*n*/*N* (%)		*n*/*N* (%)		*n*/*N* (%)	
Alopecia	141/259	(54.4)	70/132	(53.0)	71/127	(56.0)
Infection	166/349	(47.6)	80/181	(44.2)	86/168	(51.2)
Pulmonary toxicity	66/352	(18.8)	28/181	(15.5)	38/171	(22.2)
Prothrombin	17/281	(6.0)	6/149	(4.0)	11/132	(8.3)
Fever	45/353	(12.7)	26/183	(14.2)	19/170	(11.2)
Diarrhea	39/352	(11.1)	17/181	(9.4)	22/171	(12.9)
Pain	55/351	(15.7)	22/180	(12.2)	33/171	(19.3)
Nausea/vomiting	31/351	(8.8)	16/180	(8.9)	15/171	(8.8)
PCHE	3/209	(1.4)	1/110	(0.9)	2/99	(2.0)
Hypoproteinemia	30/296	(10.1)	15/151	(9.9)	15/145	(10.3)
Mucositis	22/344	(6.4)	5/177	(2.8)	17/167	(10.2)
Weight gain	43/352	(12.2)	22/183	(12.0)	21/169	(12.4)
Cardiac function	12/346	(3.5)	8/179	(4.5)	4/167	(2.4)
Bleeding	25/350	(7.1)	8/183	(4.4)	17/167	(10.2)
SGOT/SGPT	37/351	(10.5)	18/180	(10.0)	19/171	(11.1)
Central nervous system	18/349	(5.2)	5/179	(2.8)	13/170	(7.7)
Bilirubin	16/352	(4.5)	6/181	(3.3)	10/171	(5.9)
Effusion	17/350	(4.9)	8/180	(4.4)	9/170	(5.3)
Edema	21/350	(6.0)	8/179	(4.5)	13/171	(7.6)
Cutaneous toxicity	8/348	(2.3)	2/181	(1.1)	6/167	(3.6)
Alkaline phosphatase	4/306	(1.3)	2/156	(1.3)	2/150	(1.3)
Cardiac rhythm	7/344	(2.0)	3/180	(1.7)	4/164	(2.4)
Creatinine/renal toxicity	3/352	(0.9)	3/181	(1.7)	0/171	(0.0)
Obstipation	5/351	(1.4)	2/181	(1.1)	3/170	(1.8)
Hematuria	1/347	(0.3)	1/179	(0.6)	0/168	(0.0)
Extrapyramidal symptoms	4/350	(1.1)	2/180	(1.1)	2/170	(1.2)
Allergic reaction	2/346	(0.6)	1/181	(0.6)	1/165	(0.6)
Peripheral nervous system	3/351	(0.9)	2/181	(1.1)	1/170	(0.6)
Pericarditis	0/346	(0.0)	0/178	(0.0)	0/168	(0.0)

*PCHE* pseudocholinesterase, *SGOT/SGPT* serum-glutamat-oxalacetat-transaminase/serum-glutamat-pyruvat-transferase

The rate of ED was evaluated for the following time periods: days 1–14 (ED_1–14_) 3%, days 1–30 (ED_1–30_) 7%, days 1–60 (ED_1–60_) 12%, and days 1–90 (ED_1–90_) 16%. There were no statistically significant differences between the S-HAM arm (ED_1–14_: 4%, ED_1–30_: 7%, ED_1–60_: 12%, ED_1–90_: 15%) and the standard DI arm (ED_1–14_: 2%, ED_1–30_: 6%, ED_1–60_: 13%, ED_1–90_: 16%), respectively.

### Duration of critical cytopenias (2° endpoints)

**Fig. 4:**
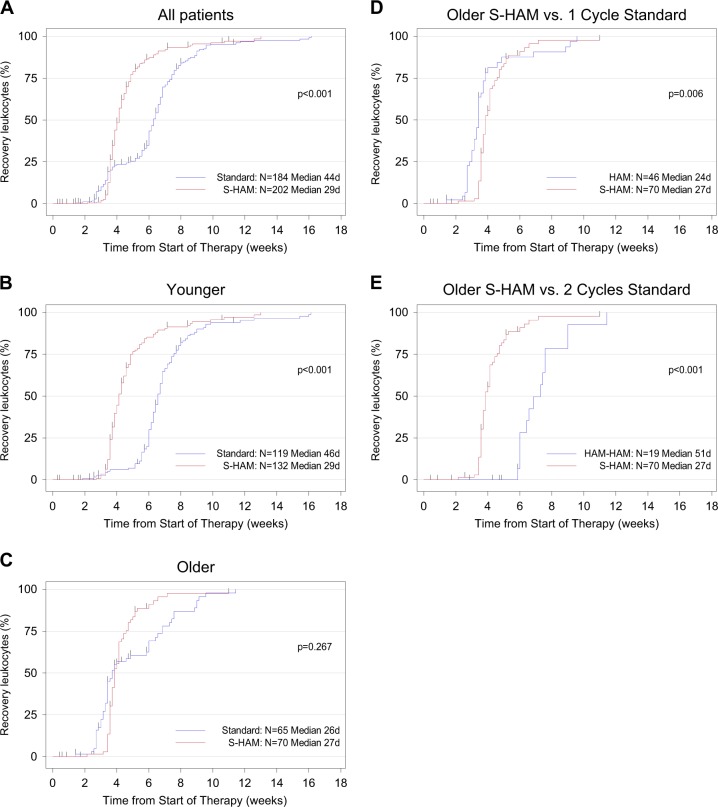
Duration of leukopenia. Duration of critical leukopenia (<1.000 leukocytes/µl) in all patients (**a**), in patients younger than <60 years (**b**), in patients older than ≥60 years (**c**). Duration of critical leukopenia was calculated from the start of therapy until recovery of peripheral blood counts and was presented as inverse Kaplan–Meier curves. In (**c**) please note the “bump” in the standard group (blue line), which is due to the fact that one subgroup of patients received only one cycle of HAM (positive selection because of adequate blast clearance in the day 16 bone marrow aspirate) and the other subgroup received two cycles of HAM (negative selection because of residual blasts in the day 16 bone marrow aspirate). Comparison of the duration of critical leukopenia (<1.000 leukocytes/µl) of all S-HAM patients older than ≥60 years versus those standard arm patients who received only one cycle of HAM (positive selection because of adequate blast clearance in the day 16 bone marrow aspirate) (**d**), of all S-HAM patients older than ≥60 years versus those standard arm patients who received two cycles of HAM (negative selection because of residual blasts in the day 16 bone marrow aspirate) (**e**)

After S-HAM, the median duration of critical leukopenia (until recovery to ≥1.000/µl leukocytes) was significantly shorter with 29 days versus 44 days after standard DI (*P* < 0.001)—Fig. 4. Within the age strata, we found critical leukopenia of 29 days after S-HAM versus 46 days after standard DI in “younger” patients (*P* < 0.001) and 27 days versus 51 days in “older” patients, if the patients had received two cycles of HAM. Similarly, the median duration of critical thrombocytopenia (46 days versus 33 days) and neutropenia (50 days versus 31 days) were also significantly reduced after S-HAM as compared to standard DI (Supplementary Figures 1 and 2). If “older” patients in the control arm had received only one cycle of therapy (=positive selection of patients) then there was no further shortening of cytopenias in the experimental arm after S-HAM (Fig. 4d, Supplementary Figures 1D, 2D).

### Duration of hospitalization (2° endpoint)

**Fig. 5:**
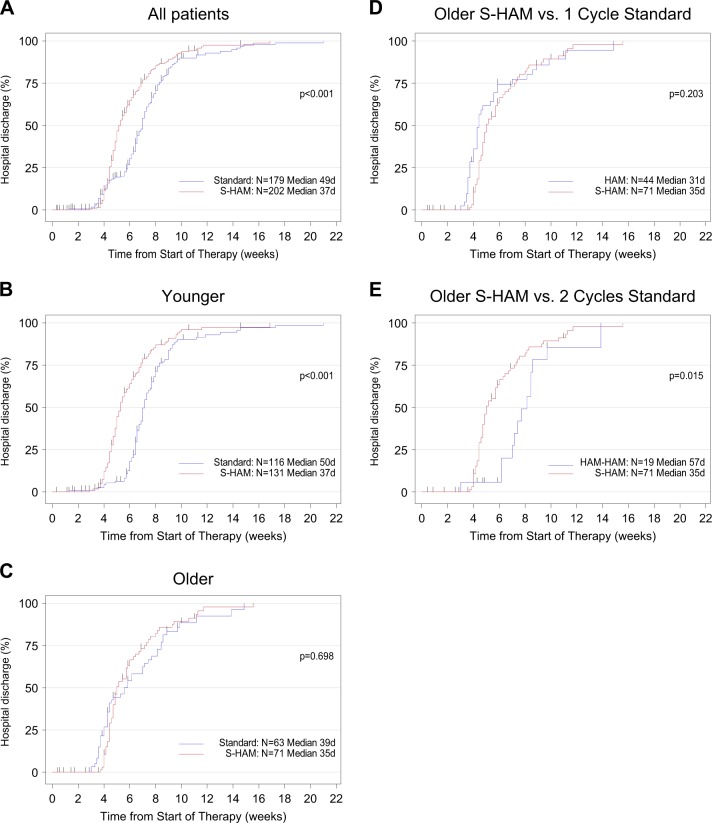
Duration of hospitalization. Duration of hospitalization in all patients (**a**), in patients younger than <60 years (**b**), in patients older than ≥60 years (**c**). Duration of hospitalization was calculated from the start of therapy until the day of discharge and was presented as inverse Kaplan–Meier curves. In (**c**) please note the “bump” in the standard group (blue line), which is due to the fact that one subgroup of patients received only one cycle of HAM (positive selection because of adequate blast clearance in the day 16 bone marrow aspirate) and the other subgroup received two cycles of HAM (negative selection because of residual blasts in the day 16 bone marrow aspirate). Comparison of the duration of hospitalization of all S-HAM patients older than ≥60 years versus those standard arm patients who received only one cycle of HAM (positive selection because of adequate blast clearance in the day 16 bone marrow aspirate) (**d**), of all S-HAM patients older than ≥60 years versus those standard arm patients who received two cycles of HAM (negative selection because of residual blasts in the day 16 bone marrow aspirate) (**e**)

For the whole group, the median duration of hospitalization (counted from day 1 of study treatment to the day of hospital discharge) was significantly shorter after S-HAM with 37 days versus 49 days after standard DI (*P* < 0.001)—Fig. 5. The respective data were 37 days versus 50 days after standard DI in “younger” patients (*P* < 0.001) and 35 days versus 57 days in “older” patients, if the patients had received two cycles of HAM.

## Conclusion

The current study evaluated whether shortening of induction therapy by the dose-dense S-HAM regimen might improve the response rate and overall prognosis of patients with newly diagnosed AML. This approach was based on promising results of a preceding trial in relapsed and refractory AML and a pilot study in previously untreated patients. The observed results revealed only a trend with a 5% higher ORR of 77% versus 72% after standard induction therapy, which was not statistically significant. This applied both for the younger group, where double induction is mandatory as well as for the older group, where double induction is only conditional—i.e., the second cycle is only applied if there were residual blasts on the day 16 bone marrow aspirate. Similarly, there was only a trend towards a longer OS with 35 months after S-HAM versus 25 months after standard DI (*P* = 0.323). No differences between both strategies were observed for the ED rate.

However, a clinically relevant and statistically significant difference was found for the duration of critical cytopenias in favor of S-HAM. Especially, critical leukopenia was reduced by a delta of 15 days from 44 days in the standard arm to 29 days after S-HAM. We attribute this phenomenon primarily to the “hematopoiesis protective timing” of treatment days 8–11 of S-HAM. This early treatment avoids the myelosuppressive effect of standard double induction, where the second cycle is started on day 21, when normal hematopoiesis is usually just starting to regenerate. The use of G-CSF following antileukemic therapy has been shown to reduce leukopenia by a median of 4 days after S-HAM induction as well as after other regimens such as high-dose AraC consolidation therapy [15, 16]. In relation to the total delta of 15 days, the differential use of G-CSF is therefore only a minor contributor. This is especially true because even though G-CSF use was mandatory after S-HAM and only optional after standard DI—nevertheless a substantial proportion of standard DI patients (17%) also received G-CSF thus making the component of differential G-CSF use even less relevant.

Even though this shortened leukopenia after S-HAM was not associated with a reduction in toxicities or in the ED rate, it nevertheless allowed a substantially earlier discharge of patients out of the hospital. The median duration of hospitalization was significantly reduced from 7 weeks (49 days) to 5 weeks (37 days). Even though these secondary endpoints were exploratory in nature and not powered to be definitive, they nevertheless precisely replicate the findings of our pilot study [10].

Our study should be considered in the context of other studies that have evaluated a “dose-dense” or “time-sequential” approach in which two cycles of intensive chemotherapy were applied during induction with either a “standard interval”—i.e., the 2nd cycle starting on day 21—or a substantially shorter interval between the cycles (“dose-dense” approach). The most convincing data were shown for pediatric AML patients in the CCG-2891 study by the Childrens’ Oncology Group COG [17] where two cycles of the DCTER regimen (Dexamethasone, Cytarabine, Thioguanine, Etoposide, Daunorubicine) were applied. After dose-dense application with a 6-day interval between cycles as compared to the 2nd cycle given only after hematologic regeneration, no increase in the CR rate was noted but long-term follow-up demonstrated a substantial prolongation of EFS and OS [18]. In adult patients, the French ALFA 9000 study [7] compared two cycles of intensive chemotherapy with the 2nd cycle given after a 4-day interval on day 8 (dose-dense approach) and a standard arm with the 2nd cycle given on day 20. In this study, only a difference in the relapse-free interval in favor of the dose-dense approach was seen. No differences were seen for the CR rate, EFS, and OS.

Our study has several limitations: (1) the hypothesis that S-HAM might improve ORR by 15% as compared to standard double induction might have been too ambitious. Hence, the number of patients recruited into the trial did not allow demonstrating a smaller though clinically relevant difference in ORR. (2) Curative AML (induction) treatment is not standardized and the regimens that we used in our study are—even though used frequently—not necessarily representative of AML induction regimens used in other parts of the world or in different study groups. (3) Genotype-specific therapy for some subgroups of AML, especially those with FLT3 mutations, is now successfully combined with intensive chemotherapy backbones [2, 19]. In how far these new agents that were used in combination with “7+3” type induction can be combined with other chemotherapy backbones like S-HAM needs to be clarified. (4) The substantially shortened duration of leukopenia and the resulting shortening of hospitalization make the S-HAM regimen an attractive option as a feasible and reliable chemotherapy backbone during induction. However, we must consider that the shortened leukopenia is pronounced only when compared to mandatory DI. Even though mandatory DI is used in many regions of the world and in many study groups [5–8, 11], its superiority to conditional DI has only been shown in historical comparisons [5, 18]. However, this question will be answered in a prospective randomized comparison by the “Dauno Double” Study of the German SAL study group which is presently recruiting.

In conclusion, the S-HAM regimen demonstrates favorable trends but no significant differences in ORR and OS as compared to standard DI. The regimen shortens critical leukopenia by more than 2 weeks with a subsequent reduction in the lengths of hospitalization from 7 to 5 weeks. In our opinion, this shorter hospital stay will appeal to patients and should also be financially favorable in most health care systems. Altogether, these features make the S-HAM regimen an attractive chemotherapy backbone for AML induction therapy.

## Electronic supplementary material

Supplementary Figure Legends

Supplementary Figure 1 - Duration of Thrombocytopenia

Supplementary Figure 2 - Duration of Neutropenia
