# Supplementary material for: Sequential high-dose cytarabine and mitoxantrone (S-HAM) versus standard double induction in acute myeloid leukemia—a phase 3 study
Source: Leukemia. 2018 Oct 1;32(12):2558–71. doi: 10.1038/s41375-018-0268-9 (PMC6286323; doi:10.1038/s41375-018-0268-9)
Supplement: Supplementary file 1 — Supplementary Figure Legends [file 41375_2018_268_MOESM1_ESM.docx]

**Supplementary Figure Legends**

**Supplementary Figure 1 – Duration of Thrombocytopenia :**

Duration of critical thrombocytopenia (< 20.000 thrombocytes/ µl) in all patients (A), in patients younger than <60 years (B), in patients older than ≥60 years (C). In (C) please note the “bump” in the standard group (blue line) which is due to the fact that one subgroup of patients received only one cycle of HAM (positive selection because of adequate blast clearance in the day 16 bone marrow aspirate) and the other subgroup received two cycles of HAM (negative selection because of residual blasts in the day 16 bone marrow aspirate). Comparison of the duration of critical thrombocytopenia (< 20.000 thrombocytes/ µl) of all S-HAM patients older than ≥60 years versus those standard arm patients who received only one cycle of HAM (positive selection because of adequate blast clearance in the day 16 bone marrow aspirate) (D), of all S-HAM patients older than ≥60 years versus those standard arm patients who received two cycles of HAM (negative selection because of residual blasts in the day 16 bone marrow aspirate) (E).

**Supplementary Figure 2 – Duration of Neutropenia:**

Duration of critical neutropenia (< 500 neutrophils/ µl) in all patients (A), in patients younger than <60 years (B), in patients older than ≥60 years (C). In (C) please note the “bump” in the standard group (blue line) which is due to the fact that one subgroup of older patients received only one cycle of HAM (positive selection because of adequate blast clearance in the day 16 bone marrow aspirate) and the other subgroup received two cycles of HAM (negative selection because of residual blasts in the day 16 bone marrow aspirate). Comparison of the duration of critical neutropenia (< 500 neutrophils/ µl) of all S-HAM patients older than ≥60 years versus those standard arm patients who received only one cycle of HAM (positive selection because of adequate blast clearance in the day 16 bone marrow aspirate) (D), of all S-HAM patients older than ≥60 years versus those standard arm patients who received two cycles of HAM (negative selection because of residual blasts in the day 16 bone marrow aspirate) (E).
