# Supplementary material for: Sequential high-dose cytarabine and mitoxantrone (S-HAM) versus standard double induction in acute myeloid leukemia—a phase 3 study
Source: Leukemia. 2018 Oct 1;32(12):2558–71. doi: 10.1038/s41375-018-0268-9 (PMC6286323; doi:10.1038/s41375-018-0268-9)
Supplement: Supplementary file 3 — Supplementary Figure 2 - Duration of Neutropenia [file 41375_2018_268_MOESM3_ESM.pptx]

## Slide 1
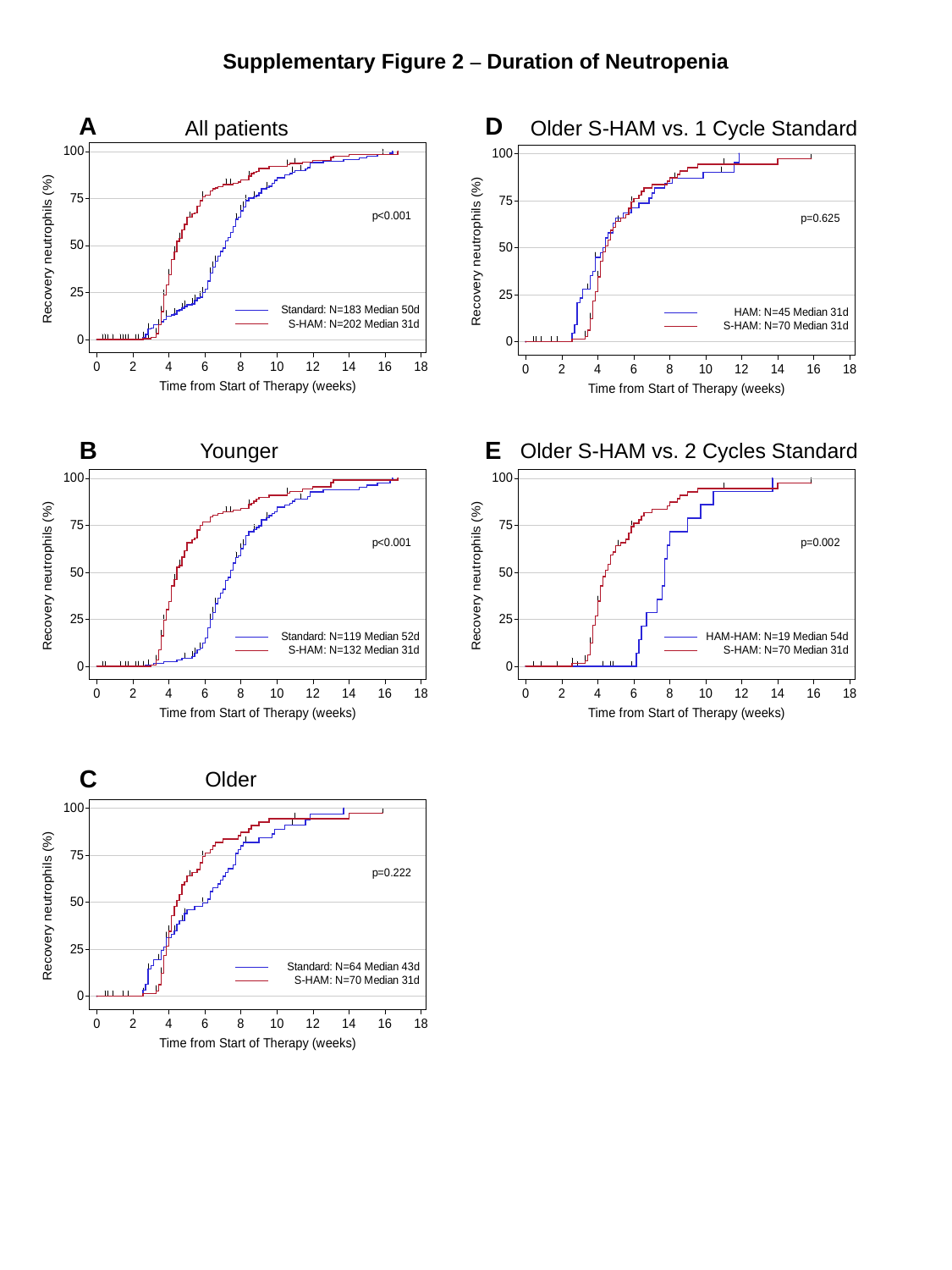

Supplementary Figure 2 ‒ Duration of Neutropenia
A
D
All patients Older S-HAM vs. 1 Cycle Standard
E
B
 Younger Older S-HAM vs. 2 Cycles Standard
C
 Older
